# Supplementary material for: Catalytic polymer self-cleavage for CO2 generation before combustion empowers materials with fire safety
Source: Nat Commun. 2024 Mar 28;15:2726. doi: 10.1038/s41467-024-46756-0 (PMC10978860; doi:10.1038/s41467-024-46756-0)
Supplement: Supplementary file 1 — Supplementary Information [file 41467_2024_46756_MOESM1_ESM.pdf]

Supplementary Information

Catalytic Polymer Self-Cleavage for CO_2 Generation Before Combustion Empowers Materials with Fire Safety

Wei Luo [1], Ming-Jun Chen [1, ✉], Ting Wang [1], Jin-Feng Feng [1], Zhi-Cheng Fu [1], Jin-Ni Deng [1], Yuan-Wei Yan [2], Yu-Zhong Wang [3], Hai-Bo Zhao [3, ✉]

[1] Green Preparation and Recycling Laboratory of Functional Polymeric Materials, College of Science, Xihua University, Chengdu, Sichuan, 610039, China

[2] Zhuzhou Times New Material Technology Co., Ltd., Zhuzhou 412007, China

[3] The Collaborative Innovation Center for Eco-Friendly and Fire-Safety Polymeric Materials, National Engineering Laboratory of Eco-Friendly Polymeric Materials (Sichuan), State Key Laboratory of Polymer Materials Engineering, College of Chemistry, Sichuan University, Chengdu, Sichuan, 610064, China

✉ Email: cmjchem@126.com; haibor7@163.com

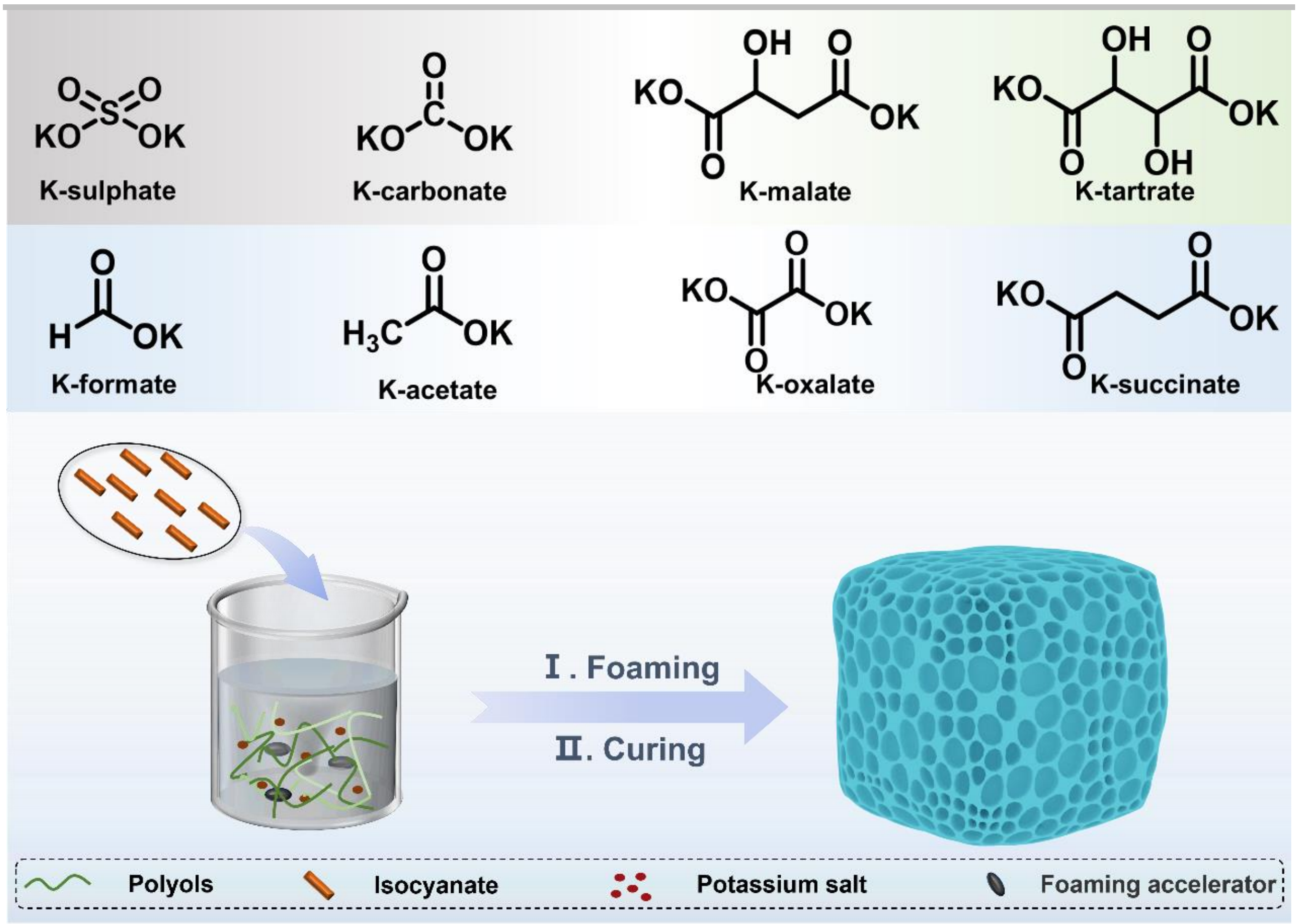

Supplementary Figure 1. Schematic diagram of the fabrication process for K-salt filled FPUF.

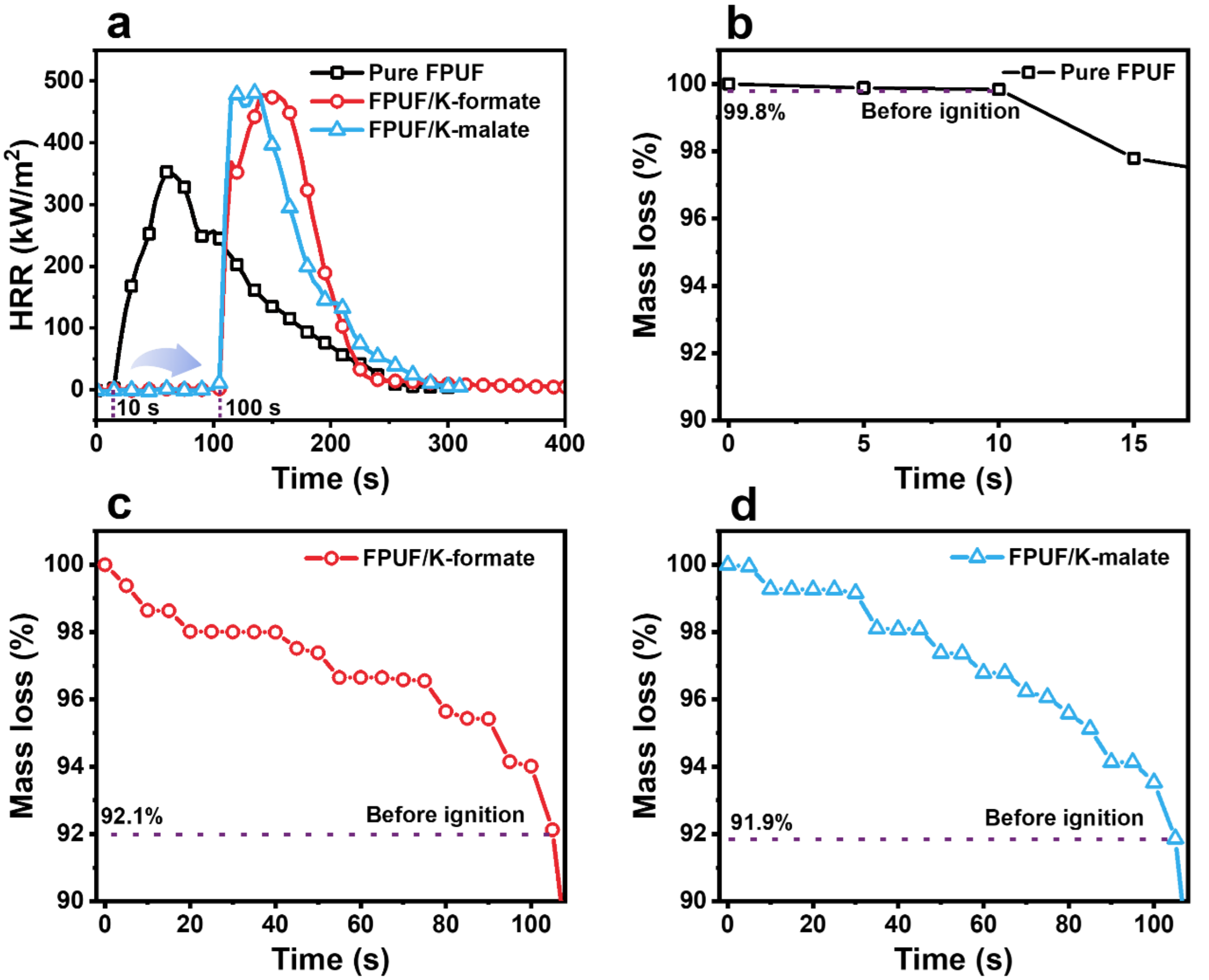

Supplementary Figure 2. a) Heat release rate (HRR) curves. The mass loss curves of b) pure FPUF, c) FPUF/K-formate, and d) FPUF/K-malate.

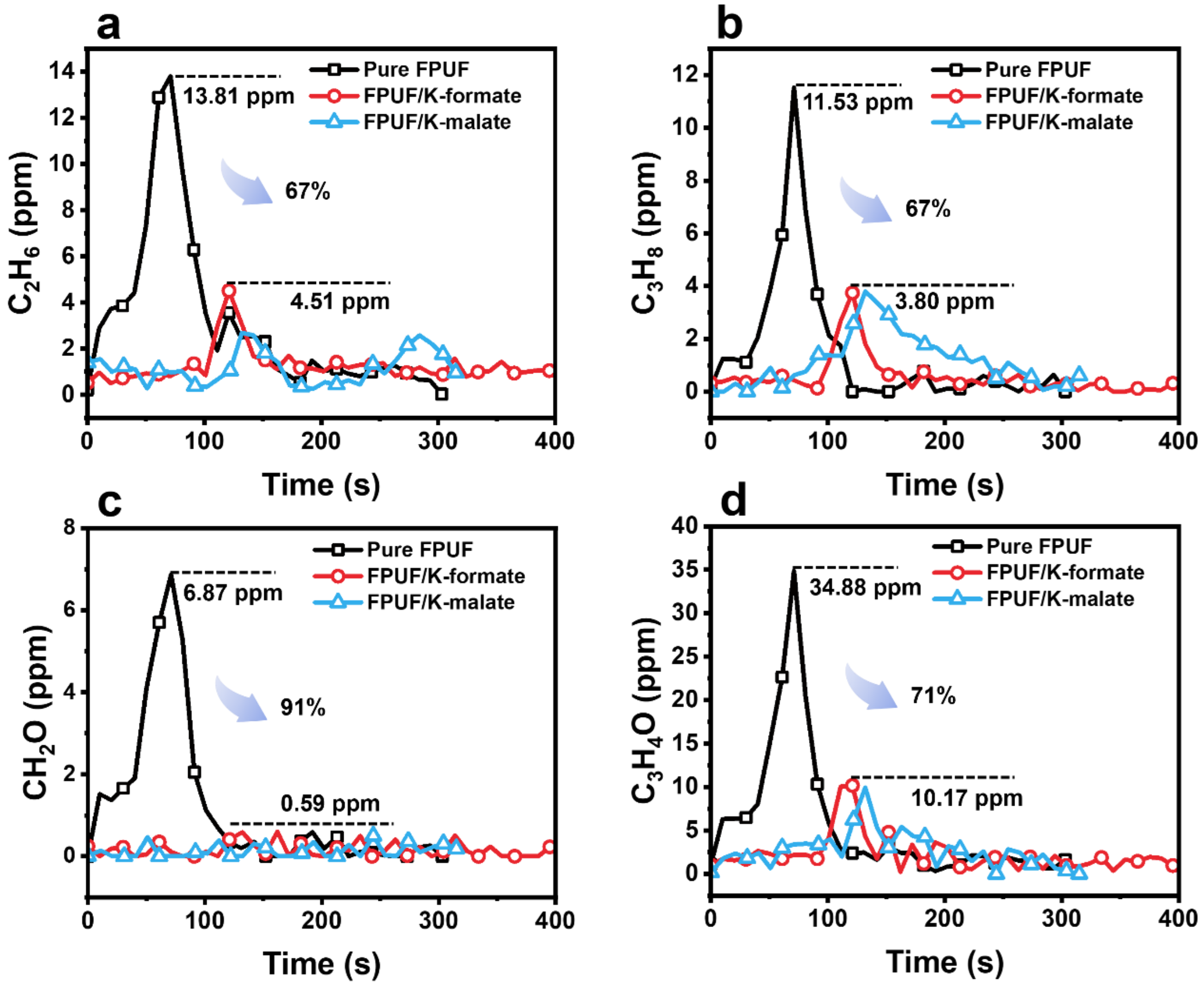

Supplementary Figure 3. The concentration of main products a) C_2H_6, b) C_3H_8, c) CH_2O, and d) C_3H_4O obtained from the cone calorimeter coupled with FTIR.

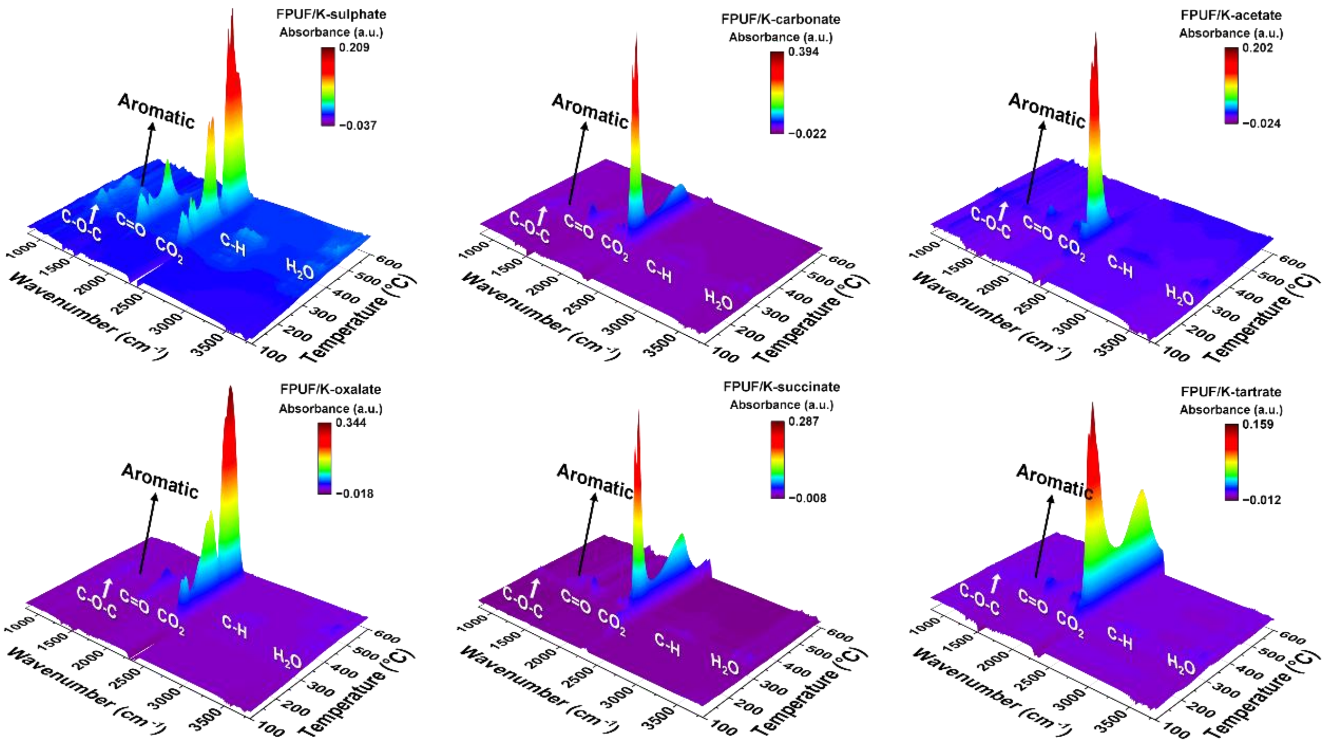

Supplementary Figure 4. The three-dimensional spectra of the pyrolysis products of K-salt filled FPUF obtained from TGA coupled with FTIR under air atmosphere.

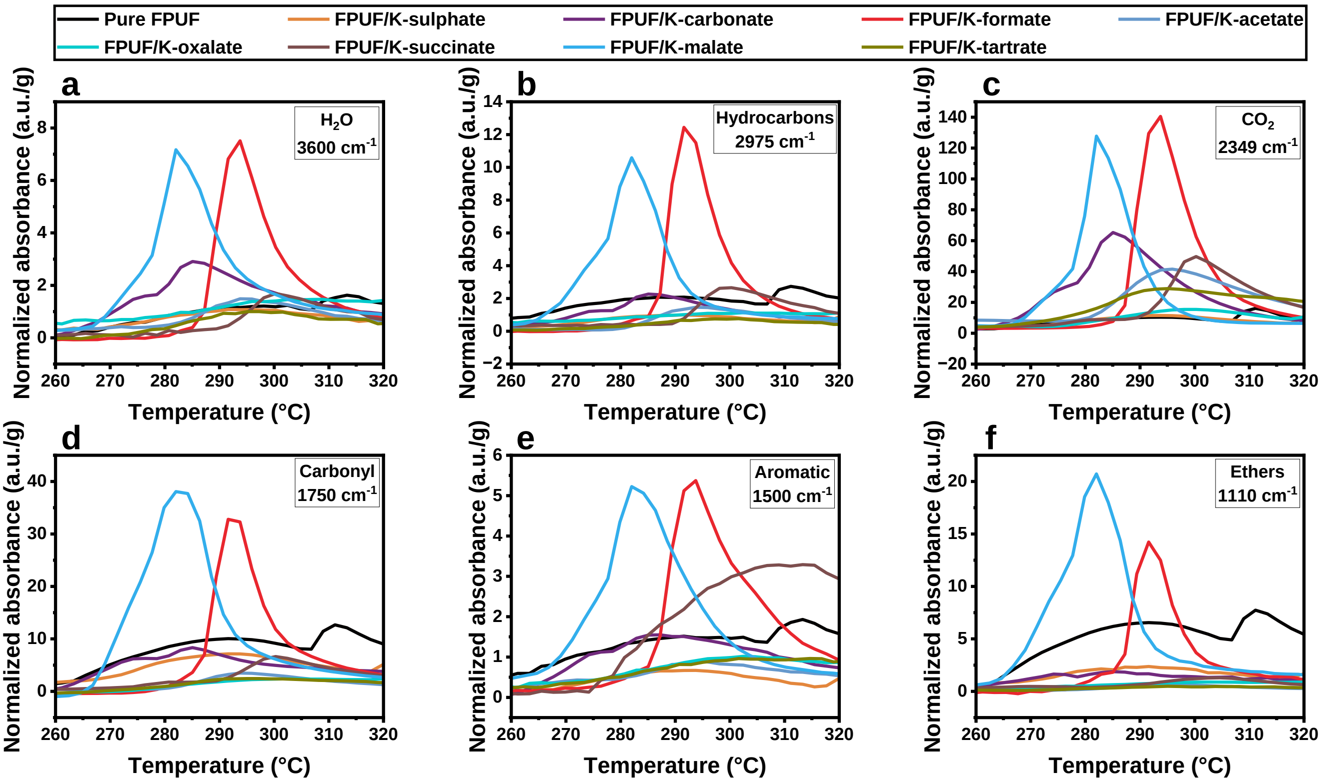

Supplementary Figure 5. Normalized absorbance curves of a) H_2O, b) hydrocarbons, c) CO_2, d) carbonyl, e) aromatic, and f) ethers during pyrolysis of K-salt filled FPUF obtained from TGA coupled with FTIR during 260-320 °C under air atmosphere. The normalized absorbance (a.u./g) of each sample is calculated by dividing the measured absorbance by its own mass.

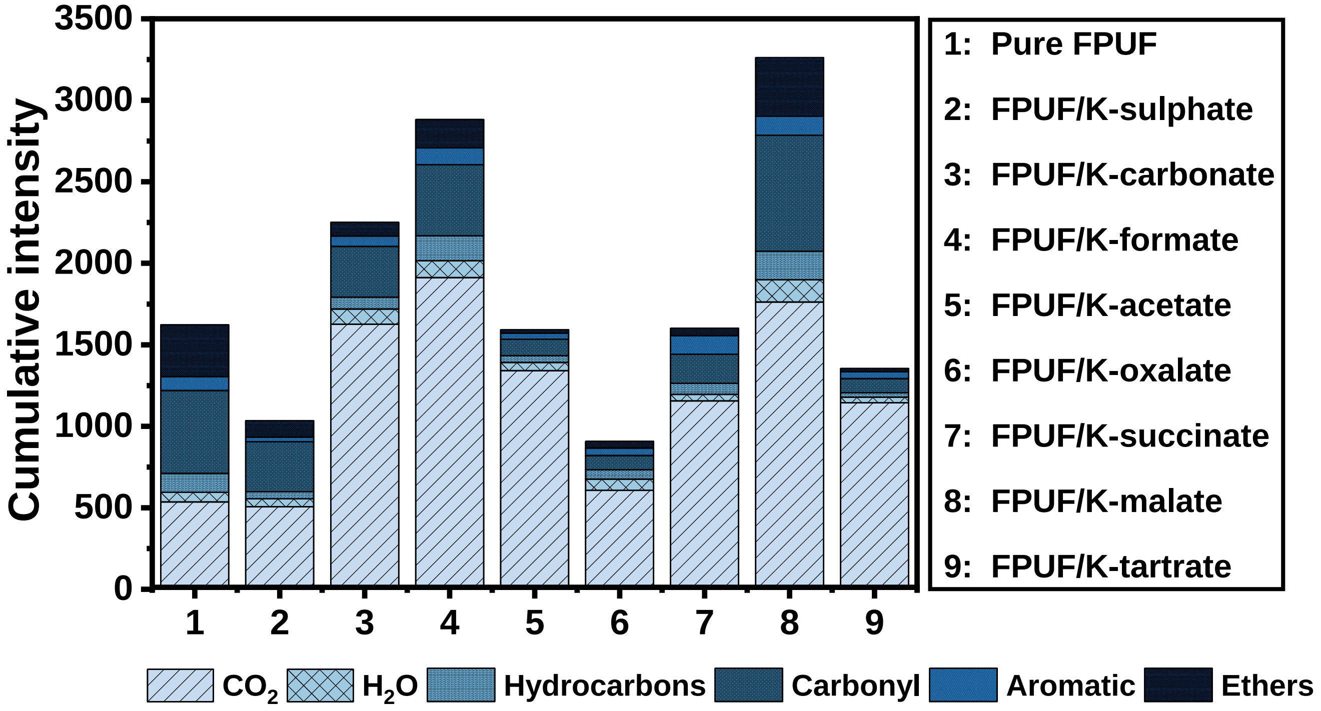

Supplementary Figure 6. Cumulative intensity of gas-phase pyrolysis products of K-salt filled FPUF during 260-320 °C under an air atmosphere. The cumulative intensity was obtained by integrating the normalized absorbance curve of K-salt filled FPUF during 260-320 °C.

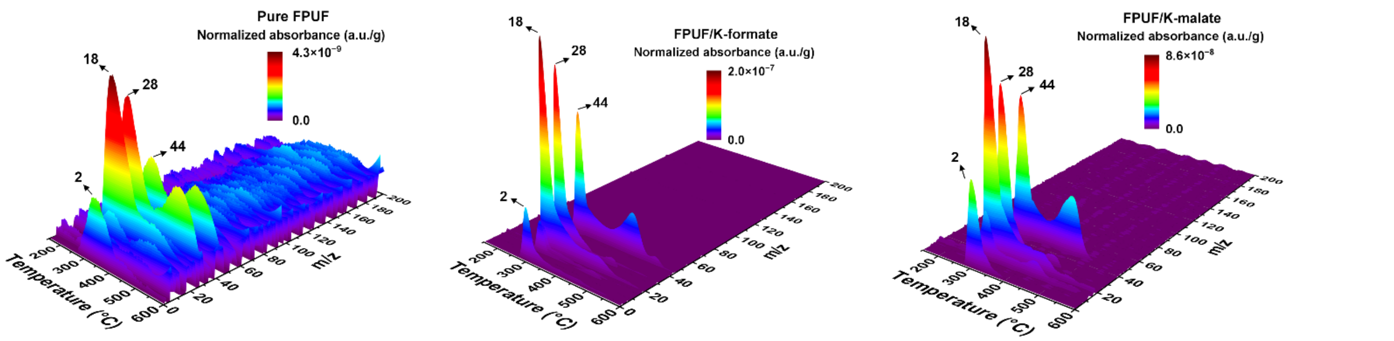

Supplementary Figure 7. Three-dimensional spectra of the pyrolysis products of pure FPUF, FPUF/K-formate, and FPUF/K-malate obtained from TGA coupled with MS under an air atmosphere. The normalized absorbance (a.u./g) of each sample is calculated by dividing the measured absorbance by its own mass.

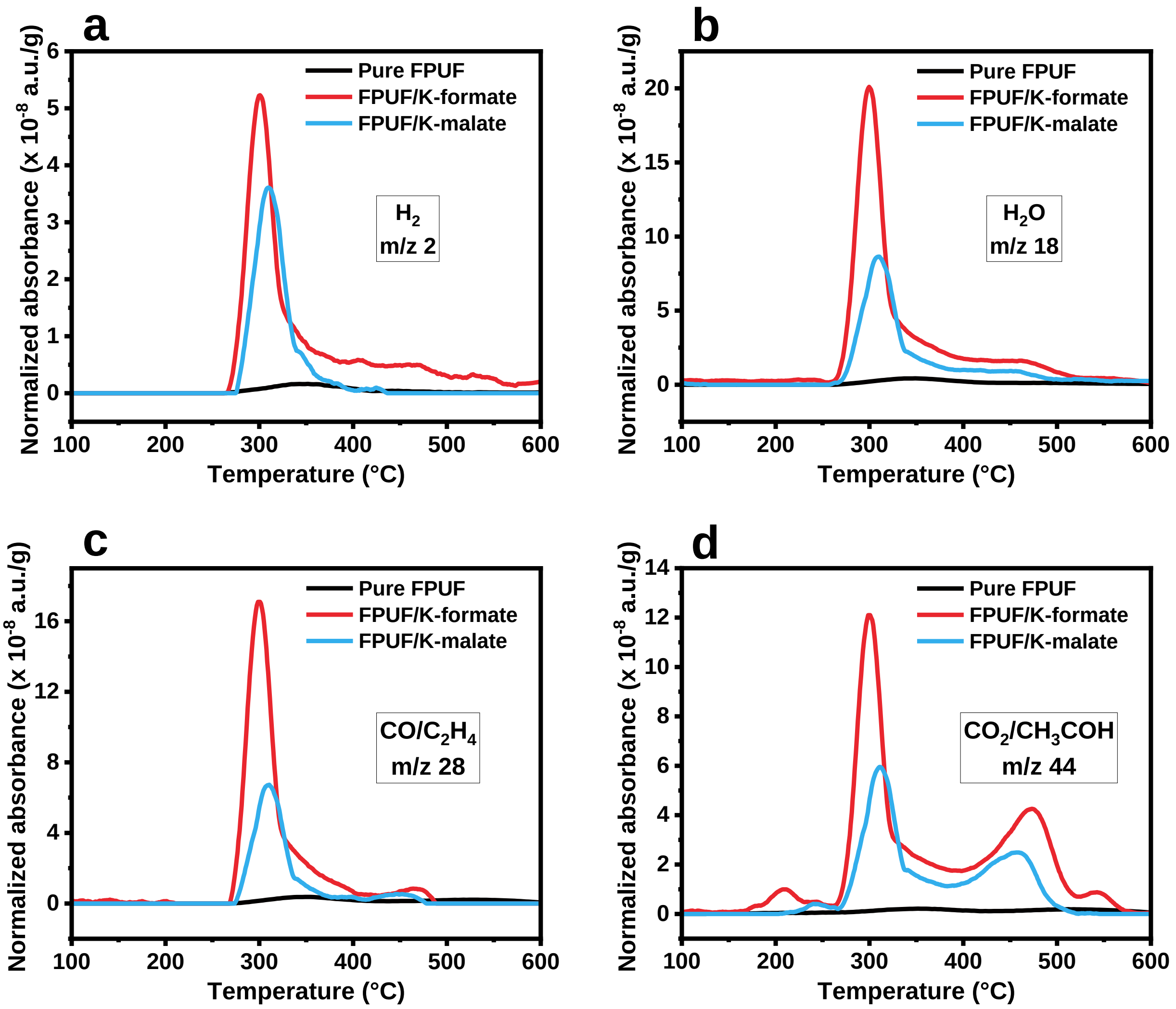

Supplementary Figure 8. Normalized absorbance curves of a) m/z 2, b) m/z 18, c) m/z 28, and d) m/z 44 during pyrolysis of K-formate and K-malate filled FPUF obtained from TGA coupled with MS under air atmosphere. The normalized absorbance (a.u./g) of each sample is calculated by dividing the measured absorbance by its own mass.

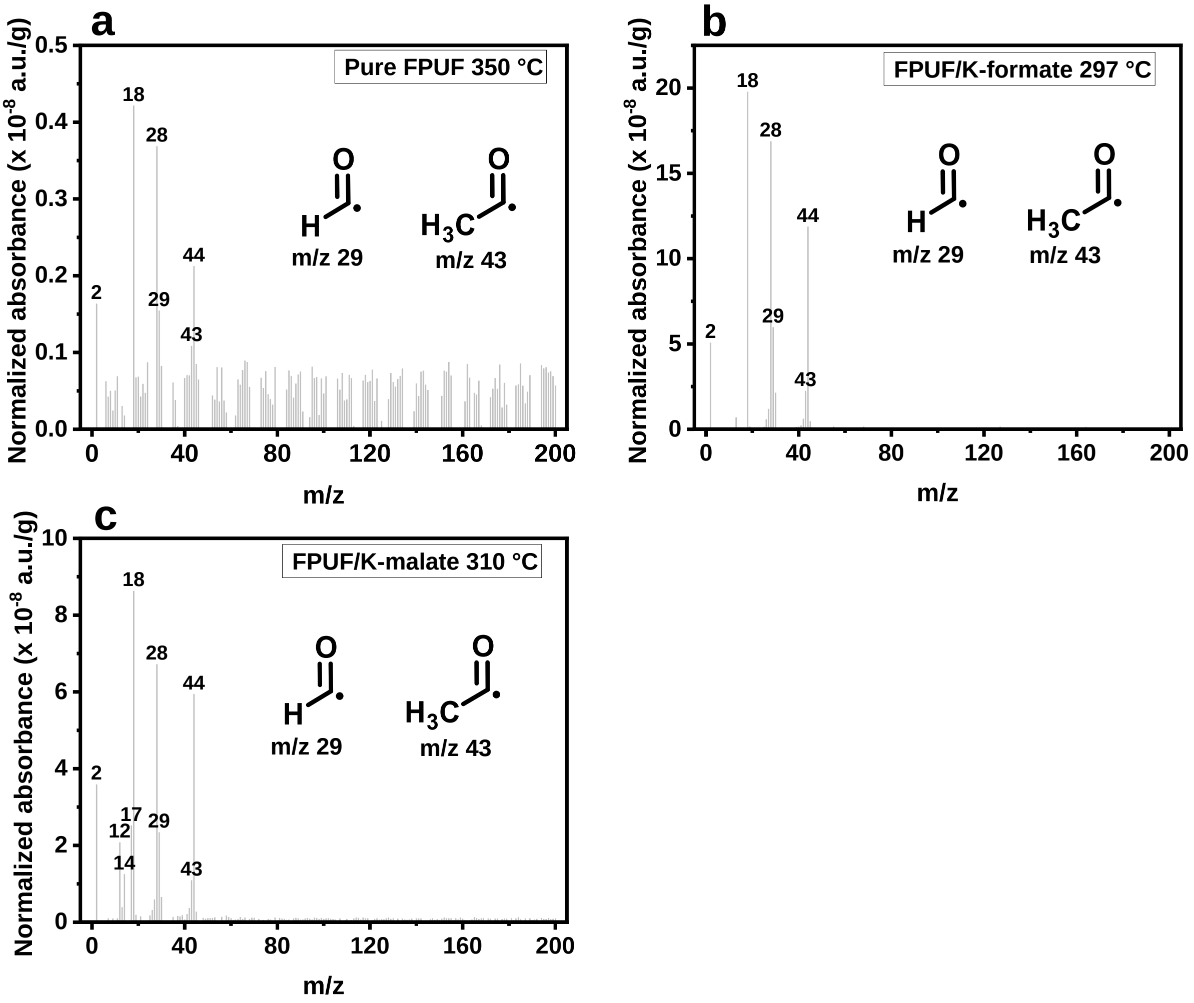

Supplementary Figure 9. Normalized absorbance curves of the pyrolysis products of a) pure FPUF, b) FPUF/K-formate, and c) FPUF/K-malate obtained from TGA coupled with MS at the maximum decomposition temperature under air atmosphere. The normalized absorbance (a.u./g) of each sample is calculated by dividing the measured absorbance by its own mass.

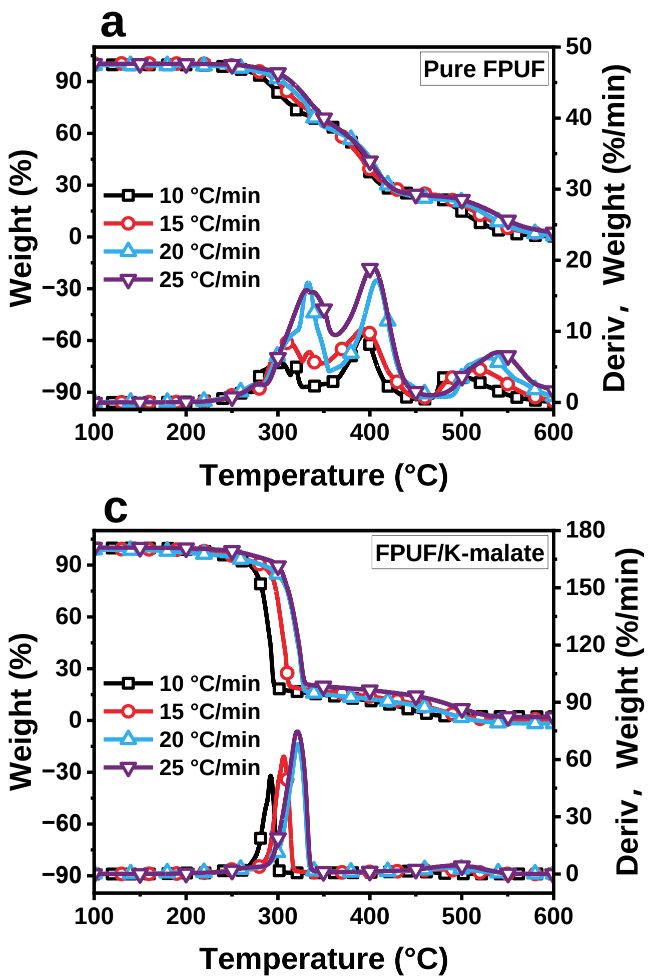

Supplementary Figure 10. The thermal decomposition curves of a) pure FPUF, b) FPUF/K-formate, and c) FPUF/K-malate at different heating rates under an air atmosphere.

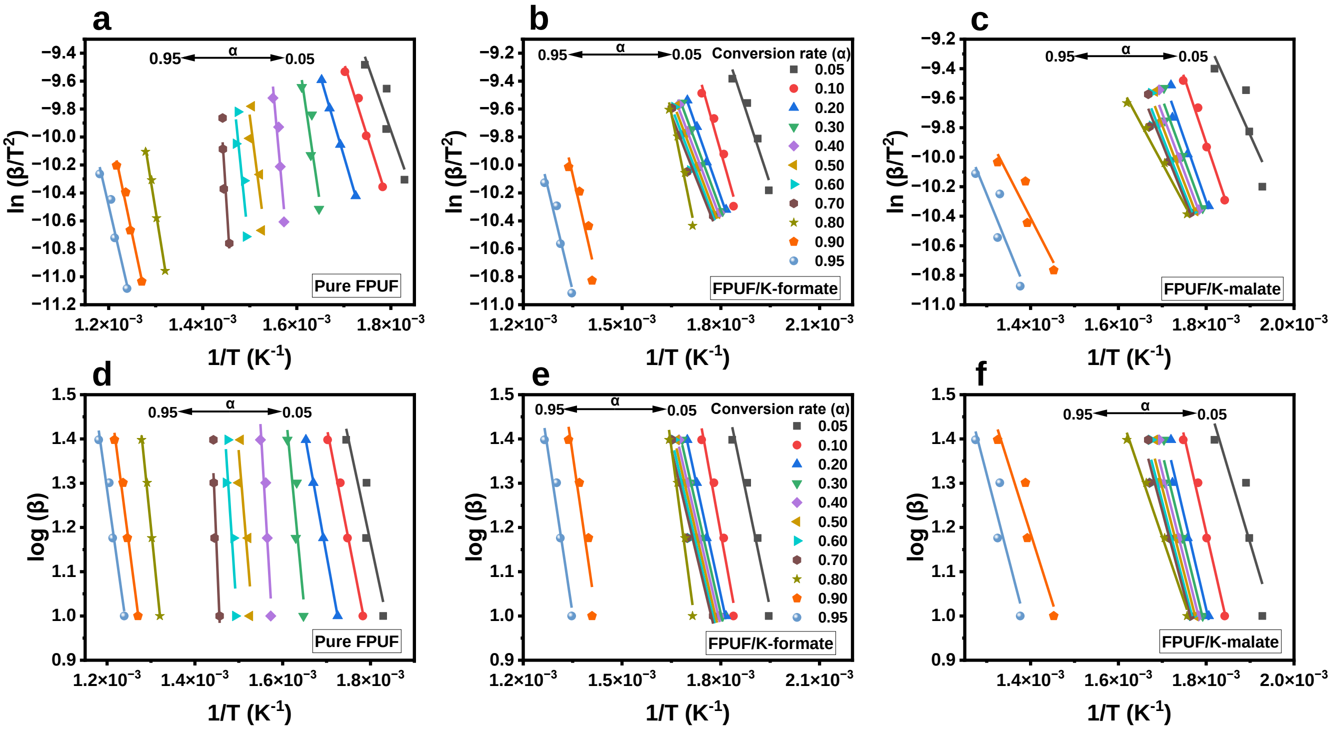

Supplementary Figure 11. Arrhenius plots of pure FPUF, FPUF/K-formate, and FPUF/K-malate with two model methods: a-c) Kissinger-Akahira-Sunose (KAS) method and d-f) Flynn-Wall-Ozawa (FWO) method.

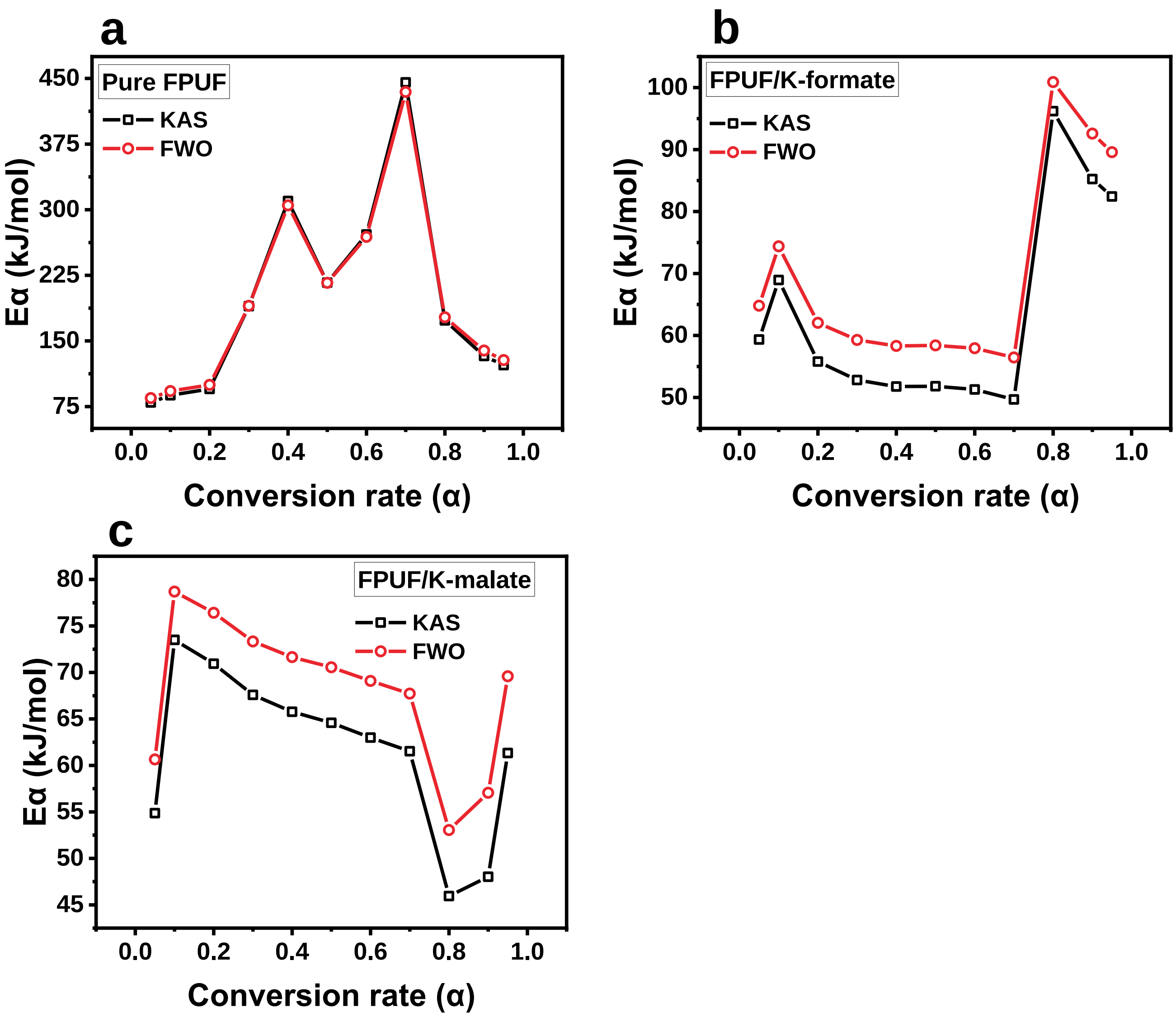

Supplementary Figure 12. Dependence of the apparent activation energy Eα and conversion rate α obtained from the KAS and FWO methods: a) pure FPUF, b) FPUF/K-formate, and c) FPUF/K-malate.

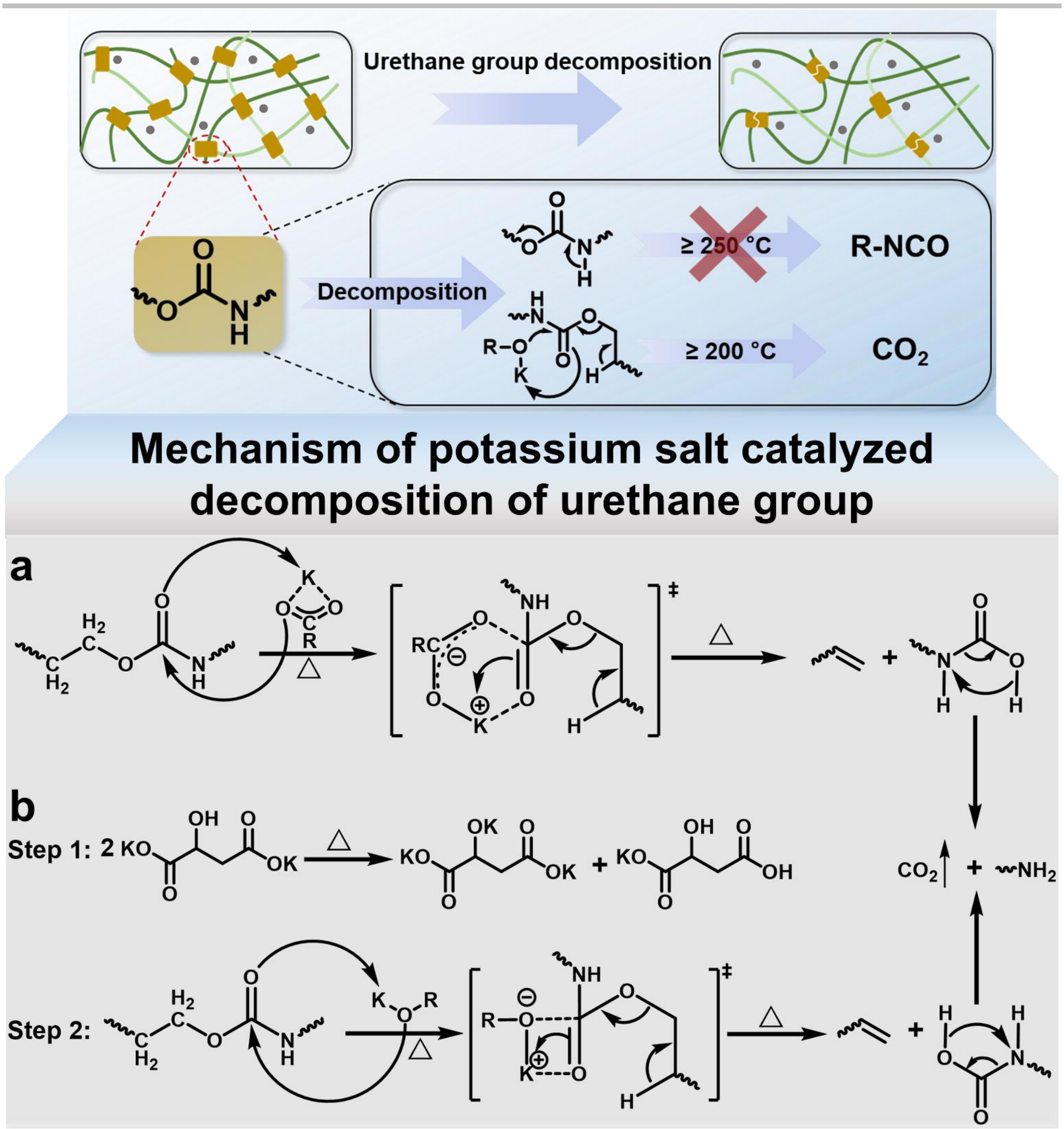

Supplementary Figure 13. Mechanism of potassium salt catalysed decomposition of the urethane group. a) Mechanism of catalytic cracking of urethane groups by hydroxyl-free potassium salts. b) Mechanism of catalytic cracking of urethane groups by hydroxyl potassium salt (taking K-malate as an example).

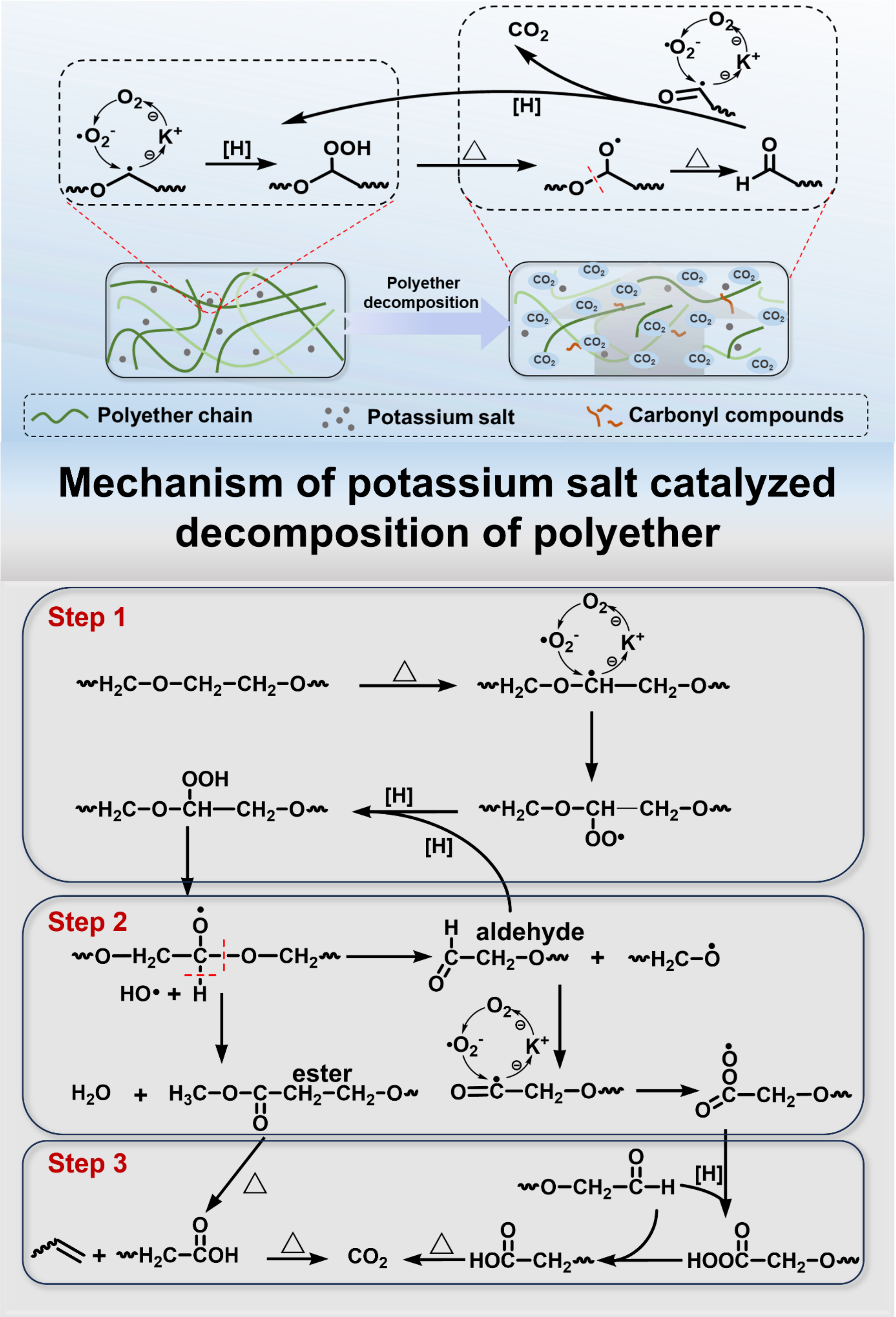

Supplementary Figure 14. Mechanism of potassium salt catalysed decomposition of polyether.

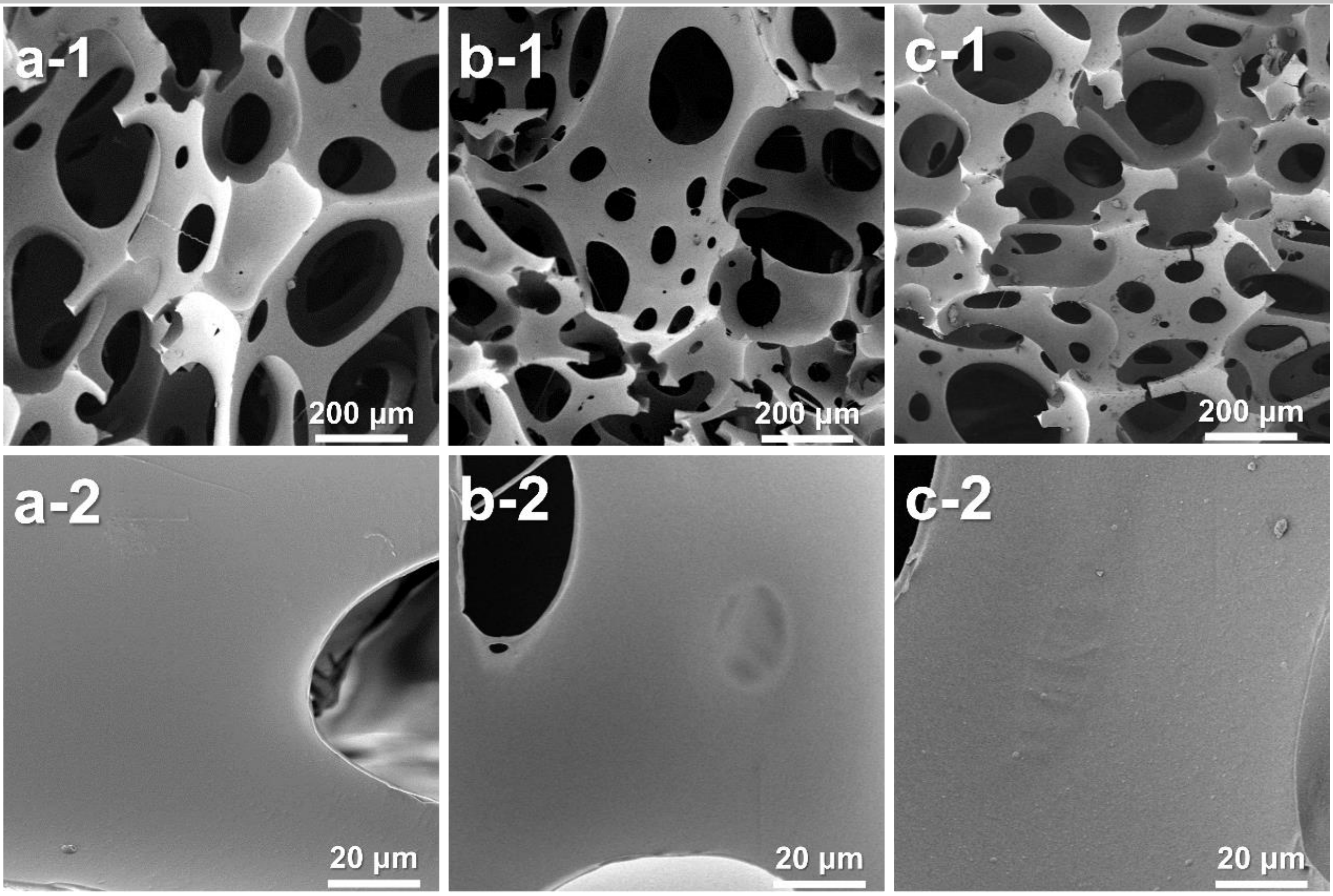

Supplementary Figure 15. Morphology of K-salt filled FPUF. SEM images for the brittle fracture of a) pure FPUF, b) FPUF/K-formate, and c) FPUF/K-malate.

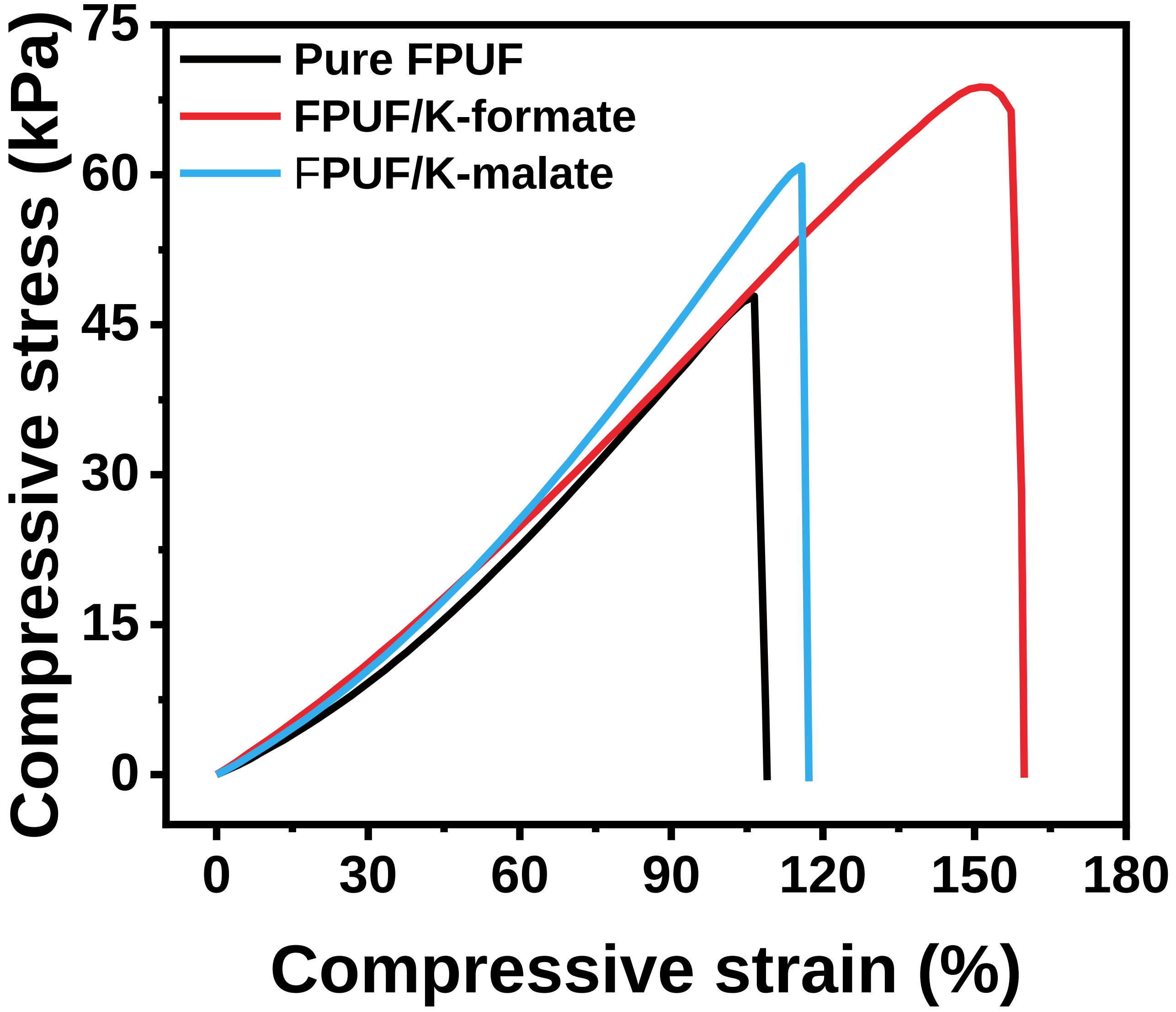

Supplementary Figure 16. Tensile stress-strain curves of pure FPUF, FPUF/K-formate, and FPUF/K-malate.

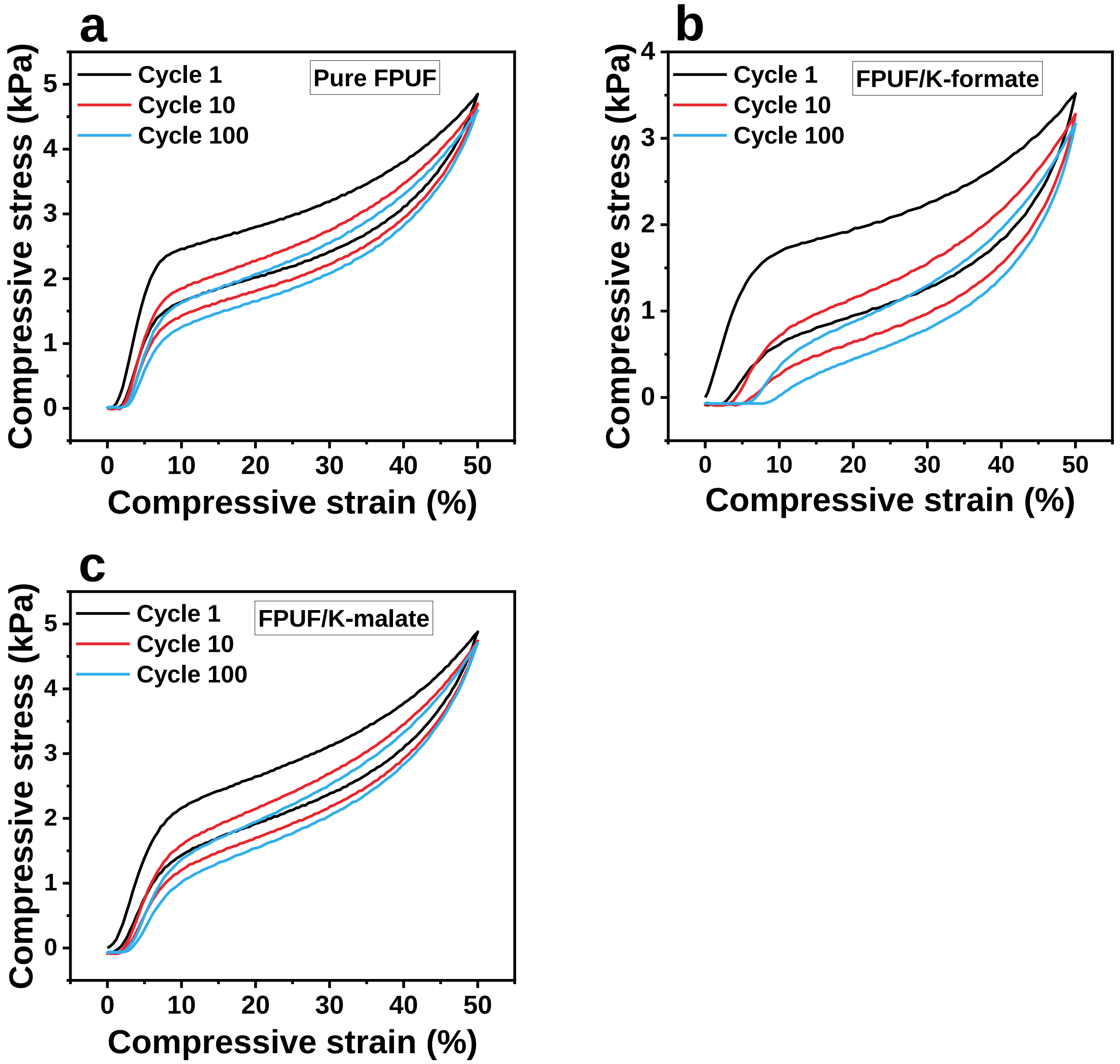

Supplementary Figure 17. Compressive cycle stress–strain curves: a) pure FPUF, b) FPUF/K-formate, and c) FPUF/K-malate.

Supplementary Table 1. Formulations of different FPUF samples.

Samples	EP-330N (wt%) [a]	H_2O (wt%)	DC-2525 (wt%)	TEOA (wt%)	A-1 (wt%)	A33B (wt%)	Filler (wt%)	MDI-2412 (wt%)	Density (kg/m³)
Pure FPUF	63.42	1.59	1.27	1.27	0.03	0.10	\	32.34	59 ± 0.5
FPUF/EG	59.63	1.49	1.19	1.19	0.02	0.09	5.96	30.41	59 ± 0.8
FPUF/TCPP	59.63	1.49	1.19	1.19	0.02	0.09	5.96	30.41	61 ± 1.0
FPUF/K-sulphate	62.72	1.57	1.25	1.25	0.03	0.09	1.09	31.99	60 ± 0.2
FPUF/K-carbonate	62.87	1.57	1.26	1.26	0.01	0.09	0.87	32.07	61 ± 0.6
FPUF/K-formate (0.01 mol)	63.13	1.58	1.26	1.26	0.01	0.03	0.53	32.19	60 ± 0.1
FPUF/K-formate (0.02 mol)	62.79	1.57	1.26	1.26	0.01	0.03	1.05	32.03	59 ± 0.2
FPUF/K-formate (0.04 mol)	62.14	1.55	1.24	1.24	0.01	0.03	2.09	31.69	61 ± 0.5
FPUF/K-acetate	62.68	1.57	1.25	1.25	0.01	0.03	1.23	31.97	61 ± 0.5
FPUF/K-oxalate	62.75	1.57	1.26	1.26	0.03	0.09	1.04	32.01	61 ± 0.2
FPUF/K-succinate	62.64	1.57	1.25	1.25	0.03	0.09	1.22	31.95	60 ± 0.2
FPUF/K-malate (0.01 mol)	63.00	1.57	1.26	1.26	0.03	0.09	0.66	32.13	60 ± 0.3
FPUF/K-malate (0.02 mol)	62.58	1.56	1.25	1.25	0.03	0.09	1.31	31.92	61 ± 0.2
FPUF/K-malate (0.04 mol)	61.60	1.54	1.23	1.23	0.02	0.37	2.59	31.42	61 ± 0.5
FPUF/K-tartrate	62.34	1.56	1.25	1.25	0.02	0.37	1.41	31.80	60 ± 0.4

Supplementary Table 2. Results of the horizontal burning test (UL 94-HB).

Samples	Burning velocity (mm/min)	Ignite absorbent cotton (Yes/No)	Rating
Pure FPUF	63.0 ± 7.6	Yes	NO
FPUF/EG	20.7 ± 3.7	No	HBF
FPUF/TCPP	/	Yes	HF-2
FPUF/K-formate	/	No	HF-1
FPUF/K-malate	/	No	HF-1

Supplementary Table 3. Performance comparison of this study with phosphorus-containing FPUF reported in recent years.

Method	Flame retardant	Loading (wt%)	LOI (%)	Variation of tensile stress (%)	Variation of tensile strain (%)	Phosphorus content of FRs (%)	Refs
Phosphorus-dominated FRs	K-formate	1.05	26.5	+42	+50	0.0	This work
	K-malate	1.31	26.0	+19	+10	0.0	This work
	PPNs	3.50	21.0	+40	+14	6.4	1
	DPM	1.80	21.8	+11	+27	13.4	2
	DOM	1.80	21.2	+17	+21	12.6	2
	DPE	1.80	21.4	+13	+30	11.8	2
	PDEO	9.00	23.0	+5	-14	21.1	3
	PSO	19.40	21.0	+15	+54	5.5	4
	BDMPP	12.05	23.0	\	\	22.2	5
	DMMP	12.11	21.5	\	\	25.0	5
	DMPMA	12.06	22.3	\	\	16.0	6
	TAMPO	12.02	19.4	\	\	10.3	6
Phosphorus and nitrogen dominated FRs	DH-DOPO	12.11	22.0	+25	-30	9.3	7
	D-Mel	12.09	24.0	+35	-11	8.6	8
	D-DICY	12.04	24.5	+53	-13	9.9	8
	D-Urea	12.02	23.5	+27	-1.6	10.6	8
	MPBT	10.00	22.5	+56	-21	8.6	9
	PPBT	10.00	21.5	+69	-29	7.3	9
	POBT	10.00	22.0	+63	-26	7.1	9
	DPPMA	9.23	23.7	+4.5	+12	9.1	10
	DMOP	16.24	22.7	-30	-13	18.7	11
	CPK	12.45	23.5	+36	-22	1.7	12

PPNs: Phosphorus containing biophenolic nanospheres; **DPM**: Hydroxymethyl diphenylphosphine oxide; **DOM**: 6-(hydroxymethyl)dibenzo[c,e][1,2]oxaphosphinine-6-oxide; **DPE**: 2-hydroxyethyl diphenylphosphinate; **POBT**: Phenyl phosphate 2-aminobenzothiazole; **PDEO**: Phosphorus-containing polyol; **PSO**: Phosphorous soybean-oil–based; **BDMPP**: Bis((dimethoxyphosphoryl)methyl) phenyl phosphate; **DMMP**: Dimethyl methanephosphonate; **DMPMA**: (Dimethoxyphosphoryl)methyl acrylate; **TAMPO**: Tris(acryloyloxymethyl)phosphine oxide; **DH-DOPO**: 9,10-dihydro-9-oxa-10-phosphaphenanthrene 10-oxide-diethanolamine; **D-Mel**: 10-hydroxy-9,10-dihydro-9-oxa-10-phosphaphenanthrene-10-oxide melamine salt; **D-DICY**: 10-hydroxy-9,10-dihydro-9-oxa-10-phosphaphenanthrene-10-oxide dicyandiamide salt; **D-Urea**: 10-hydroxy-9,10-dihydro-9-oxa-10-phosphaphenanthrene-10-oxide urea salt; **MPBT**: Methyl phosphoryl 2-aminobenzothiazole; **PPBT**: Phenylphosphoryl 2-aminobenzothiazole; **DPPMA**: Melamine salt; **DMOP**: Polyester polyol; **CPK**: Diphenylphosphine chloride modified castor oil.

Supplementary Table 4. Performance comparison of this study with other phosphorus-free flame-retardant FPUF reported in recent years.

**Flame retardant**	**Loading (wt%)**	**LOI (%)**	**Variation of tensile stress (%)**	**Variation of tensile strain (%)**	**Refs**
K-formate	1.05	26.5	+42	+50	This work
K-malate	1.31	26.0	+19	+10	This work
MEL	9.29	22.5	\	\	13
ZB	9.29	21.5	\	\	13
MH	9.29	21.5	\	\	13
LDH (Carbonate intercalated Mg/Al layered double hydroxide)	2.91	17.1	\	\	14
$Ti_3C_2T_X$	3.97	\	+48	-9.4	15
$Ti_3C_2T_X$@MOF-LDH	3.97	\	+54	+8.3	15
ZIF-8@$Ti_3C_2T_X$	3.97	\	+53	-30	16
rGO	2.02	\	+33	-27	17
rGO@CuO	2.02	\	+33	-34	17
rGO@Cu_2O	2.02	\	+67	-13	17
rGO@CSOH	2.02	\	+50	-35	17
CNS@Ce_2SnO_7	2.02	\	+86	-25	18

Supplementary Table 5. Characteristic data of different FPUF samples in the cone calorimetry.

Samples	TTI[a] (s)	pHRR[b] (kW/m^2)	t_p[c] (s)	FPI[e] (m^2s/kW)	FGI[f] ($kW/m^2/s$)
Pure FPUF	11 ± 2	389 ± 37	62 ± 3	0.03 ± 0.01	6.27 ± 0.60
FPUF/K-formate	102 ± 3	488 ± 10	138 ± 3	0.21 ± 0.01	3.54 ± 0.07
FPUF/K-malate	105 ± 1	481 ± 1	130 ± 5	0.22 ± 0.01	3.70 ± 0.01

a) TTI: Time to ignition
b) pHRR: Peak heat release rate
c) t_p: Time to pHRR
d) FPI: Fire performance index, TTI/pHRR
e) FGI: Fire growth index, pHRR/t_p

Supplementary Table 6. Peak concentrations of the main products of pure FPUF, FPUF/K-formate, and FPUF/K-malate obtained from the cone calorimeter test.

Samples	CO_2 (ppm[a])	C_2H_6 (ppm)	C_2H_6 (ppm)	CH_2O (ppm)	C_3H_4O (ppm)
Pure FPUF	8150 ± 250	13.39 ± 0.42	11.25 ± 0.28	6.68 ± 0.20	34.31 ± 0.57
FPUF/K-formate	13850 ±150	3.88 ± 0.64	3.50 ± 0.24	0.53 ± 0.06	10.76 ± 0.59
FPUF/K-malate	14000 ± 300	2.48 ± 0.22	2.95 ± 0.86	0.51 ± 0.04	9.78 ± 0.18

a) ppm: parts per million

Supplementary Table 7. Smoke toxicity gas concentration and general conventional index of toxicity (CIT_G) obtained from smoke toxicity tests for the different samples.

Samples	Pure FPUF	FPUF/TCPP	FPUF/K-formate	FPUF/K-malate
CO/(ppm)	67.8 ± 1.2	75.6 ± 9.5	67.7 ± 4.1	55.1± 6.6
HCN/(ppm)	5.1± 0.1	5.5 ± 0.3	1.9 ± 0.7	1.6 ± 0.4
NO_x/(ppm)	96.3 ± 2.8	51.3 ± 3.3	1.8 ± 0.9	12.1 ± 1.8
CIT_G	0.381 ± 0.007	0.219 ± 0.015	0.019 ± 0.003	0.053 ± 0.031

The general conventional toxicity index (CIT_G) is derived from the gaseous concentrations measured at according to the formula:

$$CIT_G = \frac{0.51\ m^3 \times 0.1\ m^2}{150\ m^3 \times 0.004225\ m^2} \times \sum_{i=1}^{i=8} \frac{c_i}{C_i}$$

where the model is the combustion of 0.1 m^2 of product; the gaseous effluents are dispersed in 150 m^3 ; the volume of the test chamber is 0.51 m^3 ; the exposed surface of the test specimen is 0.004225 m^2 . c_i is the concentration measured in mg/m^3 of the ith gas in the smoke chamber according to EN ISO 5659-2 and C_i is the IDLH reference concentration in mg/m^3 of the i th gas. CIT_G values are dimensionless. The most restrictive value of CIT_G inside the EN 45545-2 is 0.75, which means that values inferior to those could qualify the product for railway applications.

Supplementary Table 8. Results of the smoke density (Ds) test.

Samples	Ds, 1.5[a]
Pure FPUF	83.2 ± 4.7
FPUF/TCPP	107.8 ± 6.6
FPUF/K-formate	23.3 ±1.5
FPUF/K-malate	19.8 ± 1.5

a) Ds, 1.5: Smoke density at 1.5 minutes

Supplementary Table 9. TGA data of different FPUF samples under air atmosphere.

**Samples**	**$T_{5\%}$[a] (°C)**	**T_{max1}[b] (°C)**	**T_{max2} (°C)**	**CY_{260}[c] (%)**	**CY_{320} (%)**	**CY_{600} (%)**
Pure FPUF	271	290	388	96.3	70.1	0.7
FPUF/K-sulphate	278	290	394	97.1	78.1	1.6
FPUF/K-carbonate	245	186	285	92.9	17.0	1.0
FPUF/K-formate	245	184	291	93.2	8.8	1.1
FPUF/K-acetate	254	185	291	94.3	18.3	1.2
FPUF/K-oxalate	274	291	387	97.3	70.8	1.5
FPUF/K-succinate	260	195	291	95.0	15.1	0.5
FPUF/K-malate	249	193	281	92.9	15.1	1.3
FPUF/K-tartrate	274	258	293	97.0	32.0	1.0

a) $T_{5\%}$: Temperature at 5% weight loss
b) T_{max1}: Temperature at maximum thermal decomposition
c) CY_{260}: Residual rate at 260 °C
The red temperature is the T_{max} of the maximum decomposition stage of different FPUF samples.

Supplementary Table 10. Cumulative intensities of the gaseous phase pyrolysis products of K-salt filled FPUFs during 260-320 °C under an air atmosphere. The cumulative intensity was obtained by integrating the normalized absorbance curve of K-salt filled FPUF during 260-320 °C.

Samples	CO_2	H_2O	Hydrocarbons	Carbonyl	Aromatic	Ethers	Total
Pure FPUF	536	59	116	509	84	318	1622
FPUF/K-sulphate	507	47	44	306	28	102	1034
FPUF/K-carbonate	1626	93	77	311	64	83	2254
FPUF/K-formate	1912	103	153	436	104	174	2882
FPUF/K-acetate	1341	50	42	100	37	22	1592
FPUF/K-oxalate	607	69	57	88	45	42	908
FPUF/K-succinate	1156	40	69	177	114	45	1601
FPUF/K-malate	1762	136	176	711	118	359	3262
FPUF/K-tartrate	1144	34	27	87	42	20	1354

Supplementary Table 11. Activation energy Eα and R^2 of pure FPUF, FPUF/K-formate, and FPUF/K-malate sludge at different conversion degrees.

			**KAS[a]**			
**α[c]**	**Pure FPUF**		**FPUF/K-formate**		**FPUF/K-malate**	
	**Eα[d] (kJ/mol)**	**[e]R^2**	**Eα (kJ/mol)**	**R^2**	**Eα (kJ/mol)**	**R^2**
0.05	79.68	0.8412	59.34	0.9447	54.86	0.7483
0.10	87.95	0.9783	68.94	0.9551	73.50	0.9792
0.20	95.06	0.9998	55.79	0.9936	70.94	0.9450
0.30	189.70	0.8204	52.81	0.9803	67.59	0.9409
0.40	309.97	0.9043	51.75	0.9733	65.77	0.9432
0.50	216.61	0.7570	51.80	0.9673	64.58	0.9449
0.60	271.64	0.7665	51.29	0.9547	62.99	0.9394
0.70	445.51	0.8440	49.68	0.9395	61.52	0.9445
0.80	173.38	0.9940	96.19	0.9693	45.94	0.9838
0.90	132.81	0.9761	85.24	0.8599	48.02	0.8609
0.95	122.36	0.9500	82.43	0.9319	61.33	0.8042
			**FWO[b]**			
**α**	**Pure FPUF**		**FPUF/K-formate**		**FPUF/K-malate**	
	**Eα (kJ/mol)**	**R^2**	**Eα (kJ/mol)**	**R^2**	**Eα (kJ/mol)**	**R^2**
0.05	84.61	0.8688	64.79	0.9580	60.64	0.8013
0.10	92.72	0.9824	74.38	0.9651	78.70	0.9837
0.20	99.74	0.9998	62.04	0.9952	76.42	0.9565
0.30	190.09	0.8354	59.27	0.9855	73.33	0.9539
0.40	304.89	0.9101	58.31	0.9805	71.65	0.9561
0.50	216.43	0.7748	58.40	0.9761	70.56	0.9576
0.60	268.97	0.7807	57.94	0.9670	69.09	0.9537
0.70	434.56	0.8505	56.45	0.9563	67.72	0.9580
0.80	177.04	0.9948	100.89	0.9748	53.05	0.9894
0.90	138.99	0.9803	92.57	0.8894	57.05	0.9071
0.95	128.05	0.9595	89.57	0.9486	69.58	0.8445
			**Average**			
**α**	**Pure FPUF**		**FPUF/K-formate**		**FPUF/K-malate**	
	**Eα (kJ/mol)**	**R^2**	**Eα (kJ/mol)**	**R^2**	**Eα (kJ/mol)**	**R^2**
0.05	82.15	-	62.07	-	57.75	-
0.10	90.34	-	71.66	-	76.10	-
0.20	97.40	-	58.92	-	73.68	-
0.30	189.90	-	56.04	-	70.46	-
0.40	307.43	-	55.03	-	68.71	-
0.50	216.52	-	55.10	-	67.57	-
0.60	270.31	-	54.62	-	66.04	-
0.70	440.04	-	53.07	-	64.62	-
0.80	175.21	-	98.54	-	49.50	-
0.90	135.90	-	88.91	-	52.54	-
0.95	125.21	-	86.00	-	65.46	-

a) KAS: Kissinger-Akahira-Sunose model
b) FWO: Flynn-Wall-Ozawa model
c) α: Conversion rate
d) Eα: The apparent activation energy of different conversion rates
e) R^2: The correlation coefficient of the linear relationship

Supplementary Table 12. Mechanical properties of pure FPUF, FPUF/K-formate, and FPUF/K-malate

Samples	Pure FPUF	FPUF/K-formate	FPUF/K-malate
Density (kg/m^3)	59 ± 0.5	59 ± 0.2	61 ± 0.2
Tensile strength (kPa)	48 ± 6	68 ± 4	57 ± 4
Elongation at break (%)	105 ± 5	157 ± 7	115 ± 6
Tensile toughness (kJ/m^2)	25 ± 3	52 ± 5	35 ± 4
Compressive strength (kPa)	4.9	3.5	4.9
Compressive strength recovery rate (%)	94.9	90.3	96.5
Compressive deformation recovery rate (%)	99.6	90.0	93.0

Supplementary References

1. Zeng, F.R. et al. Recyclable Biophenolic Nanospheres for Sustainable and Durable Multifunctional Applications in Thermosets. *ACS Materials Letters* **5**, 1692-1702 (2023).
2. Zeng, F.R. et al. Molecular-micron multiscale toughening and flame retarding for polyurethane foams. *Chemical Engineering Journal* **454,** 140023 (2023).
3. Rao, W. et al. A reactive phosphorus-containing polyol incorporated into flexible polyurethane foam: Self-extinguishing behavior and mechanism. *Polymer Degradation and Stability* **153**, 192-200 (2018).
4. Chan, Y.Y., Ma, C., Zhou, F., Hu, Y. & Schartel, B. Flame retardant flexible polyurethane foams based on phosphorous soybean-oil polyol and expandable graphite. *Polymer Degradation and Stability* **191**, 109656 (2021).
5. Zhou, F. et al. Synthesis of a novel liquid phosphorus-containing flame retardant for flexible polyurethane foam: Combustion behaviors and thermal properties. *Polymer Degradation and Stability* **171**, 109029 (2020).
6. Ma, C. et al. Fabrication of flexible polyurethane/phosphorus interpenetrating polymer network (IPN) foam for enhanced thermal stability, flame retardancy and mechanical properties. *Polymer Degradation and Stability* **189**, 109602 (2021).
7. Yin, Z., Chu, F., Yu, B., Wang, B. & Hu, Y. Hierarchical Ti(3)C(2)T(x)@BPA@PCL for flexible polyurethane foam capable of anti-compression, self-extinguishing and flame-retardant. *J Colloid Interface Sci* **626**, 208-220 (2022).
8. Ma, S.C. et al. Effects of novel phosphorus-nitrogen-containing DOPO derivative salts on mechanical properties, thermal stability and flame retardancy of flexible polyurethane foam. *Polymer Degradation and Stability* **177**, 109160 (2020).
9. Zhang, S. et al. The improvement of fire safety performance of flexible polyurethane foam by Highly-efficient P-N-S elemental hybrid synergistic flame retardant. *J Colloid Interface Sci* **606**, 768-783 (2022).
10. Rao, W.H. et al. Flame-Retardant Flexible Polyurethane Foams with Highly Efficient Melamine Salt. *Industrial & Engineering Chemistry Research* **56**, 7112-7119 (2017).
11. Rao, W. et al. Persistently flame-retardant flexible polyurethane foams by a novel phosphorus-containing polyol. *Chemical Engineering Journal* **343**, 198-206 (2018).
12. Zhang, S.H. et al. High-performance flexible polyurethane from renewable castor oil: Preparation, properties

and mechanism. *Composites Part A: Applied Science and Manufacturing* **159**, 107034 (2022).

13. Wang, C.-Q., Lv, H.-N., Sun, J. & Cai, Z.-S. Flame retardant and thermal decomposition properties of flexible polyurethane foams filled with several halogen-free flame retardants. *Polymer Engineering & Science* **54**, 2497-2507 (2014).
14. Gómez Fernández, S., Günther, M., Schartel, B., Corcuera, M.A. & Eceiza, A. Impact of the combined use of layered double hydroxides, lignin and phosphorous polyol on the fire behavior of flexible polyurethane foams. *Industrial Crops and Products* **125**, 346-359 (2018).
15. Zhou, Y. et al. MOF-derived 3D petal-like CoNi-LDH array cooperates with MXene to effectively inhibit fire and toxic smoke hazards of FPUF. *Chemosphere* **297**, 134134 (2022).
16. Yin, Z. et al. Functionalizing Ti3C2Tx for enhancing fire resistance and reducing toxic gases of flexible polyurethane foam composites with reinforced mechanical properties. *Journal of Colloid and Interface Science* **607**, 1300-1312 (2022).
17. Jia, P. et al. Design of copper salt@graphene nanohybrids to accomplish excellent resilience and superior fire safety for flexible polyurethane foam. *Journal of Colloid and Interface Science* **606**, 1205-1218 (2022).
18. Yu, X. et al. Effects of graphene nanosheets decorated by cerium stannate on the enhancement of flame retardancy and mechanical performances of flexible polyurethane foam composites. *Polymers for Advanced Technologies* **33**, 290-302 (2021).
