# Supplementary material for: Catalytic polymer self-cleavage for CO2 generation before combustion empowers materials with fire safety
Source: Nat Commun. 2024 Mar 28;15:2726. doi: 10.1038/s41467-024-46756-0 (PMC10978860; doi:10.1038/s41467-024-46756-0)
Supplement: Supplementary file 3 — Description of Additional Supplementary Files [file 41467_2024_46756_MOESM3_ESM.pdf]

Description of Additional Supplementary Files

File Name: Supplementary Movie 1
Description: K-formate filled FPUF(oxygen index 26.5%)

File Name: Supplementary Movie 2
Description: K-malate filled FPUF (oxygen index 26.0%)

File Name: Supplementary Movie 3
Description: K-formate filled FPUF(oxygen index 30.5%)

File Name: Supplementary Movie 4
Description: Horizontal burning test of pure FPUF

File Name: Supplementary Movie 5
Description: Horizontal burning test of EG filled FPUF

File Name: Supplementary Movie 6
Description: Horizontal burning test of TCPP filled FPUF

File Name: Supplementary Movie 7
Description: Horizontal burning test of K-formate filled FPUF

File Name: Supplementary Movie 8
Description: Horizontal burning test of K-malate filled FPUF
